# A novel class of fast‐acting antimalarial agents: Substituted 15‐membered azalides

**DOI:** 10.1111/bph.15292

**Published:** 2020-12-16

**Authors:** Mihaela Peric, Dijana Pešić, Sulejman Alihodžić, Andrea Fajdetić, Esperanza Herreros, Francisco Javier Gamo, Iñigo Angulo‐Barturen, María Belén Jiménez‐Díaz, Santiago Ferrer‐Bazaga, María S. Martínez, Domingo Gargallo‐Viola, Amanda Mathis, Albane Kessler, Mihailo Banjanac, Jasna Padovan, Vlatka Bencetić Mihaljević, Vesna Munic Kos, Mirjana Bukvić, Vesna Eraković Haber, Radan Spaventi

**Affiliations:** ^1^ GlaxoSmithKline Research Centre Zagreb Ltd. Zagreb Croatia; ^2^ GlaxoSmithKline, Tres Cantos Medicines Development Campus, Diseases of the Developing World Tres Cantos (Madrid) Spain; ^3^ GlaxoSmithKline Research Triangle Park North Carolina USA; ^4^ Center for Translational and Clinical Research, Department for Intercellular Communication University of Zagreb School of Medicine Zagreb Croatia; ^5^ Fidelta Ltd. Zagreb Croatia; ^6^ The Art of Discovery Bizkaia Basque Country Spain; ^7^ ABAC Therapeutics Barcelona Spain; ^8^ BioCryst Pharmaceuticals Durham North Carolina USA; ^9^ Department of Physiology and Pharmacology Karolinska Institutet Stockholm Sweden; ^10^ Triadelta Partners Ltd Zagreb Croatia; ^11^ Medicines for Malaria Venture Geneva 15 Switzerland

**Keywords:** antimalarial, azalide, *in vivo* efficacy, macrolide, malaria, mode of action, pharmacokinetics

## Abstract

**Background and Purpose:**

Efficacy of current antimalarial treatments is declining as a result of increasing antimalarial drug resistance, so new and potent antimalarial drugs are urgently needed. Azithromycin, an azalide antibiotic, was found useful in malaria therapy, but its efficacy in humans is low.

**Experimental Approach:**

Four compounds belonging to structurally different azalide classes were tested and their activities compared to azithromycin and chloroquine. in vitro evaluation included testing against sensitive and resistant *Plasmodium falciparum* , cytotoxicity against HepG2 cells, accumulation and retention in human erythrocytes, antibacterial activity, and mode of action studies (delayed death phenotype and haem polymerization). in vivo assessment enabled determination of pharmacokinetic profiles in mice, rats, dogs, and monkeys and in vivo efficacy in a humanized mouse model.

**Key Results:**

Novel fast‐acting azalides were highly active in vitro against *P. falciparum* strains exhibiting various resistance patterns, including chloroquine‐resistant strains. Excellent antimalarial activity was confirmed in a *P. falciparum* murine model by strong inhibition of haemozoin‐containing trophozoites and quick clearance of parasites from the blood. Pharmacokinetic analysis revealed that compounds are metabolically stable and have moderate oral bioavailability, long half‐lives, low clearance, and substantial exposures, with blood cells as the preferred compartment, especially infected erythrocytes. Fast anti‐plasmodial action is achieved by the high accumulation into infected erythrocytes and interference with parasite haem polymerization, a mode of action different from slow‐acting azithromycin.

**Conclusion and Implications:**

The hybrid derivatives described here represent excellent antimalarial drug candidates with the potential for clinical use in malaria therapy.

**Abbreviations**
DMPK: drug metabolism and pharmacokinetics
F: bioavailability
LBF: liver blood flow
PK/PD: pharmacokinetic/pharmacodynamics

**What is already known**

- New antimalarials are urgently needed to eradicate malaria, one of the most devastating infectious diseases.
- Ideal antimalarial characteristics include fast‐acting, resistant parasites coverage, orally available, no relapse and single‐dose efficacy.

**What this study adds**

- A novel class of antimalarial hybrid molecules with improved performance over starting azithromycin and chloroquine.
- These derivatives show characteristics consistent with ideal new antimalarial compounds.

**What is the clinical significance**

- These compounds could be used for treatment and/or prophylaxis of malaria.
- Their pharmacological properties ensure affordability for patients in developing countries.

## 1 INTRODUCTION

Malaria incidence continues to overwhelm the tropics with estimated 228 million new infections and almost 405,000 deaths in 2018, with antimalarial drug resistance additionally hindering control of the disease (WHO, 2019). Of the five *Plasmodium* species infecting humans, *Plasmodium falciparum* is the leading cause of mortality, and its decreasing susceptibility to current antimalarial drugs increases the risk of inadequate therapy (Ashley et al., 2014; Fairhurst et al., 2012; Imwong et al., 2017; Menard & Dondorp, 2017). Even though prevention and control efforts produced measurable effects on public health, new management tools and intensified drug discovery efforts are crucial in controlling malaria and expanding available treatment options (Anthony, Burrows, Duparc, Moehrle, & Wells, 2012; Wells, Hooft van, & Van Voorhis, 2015).

**FIGURE 1:**
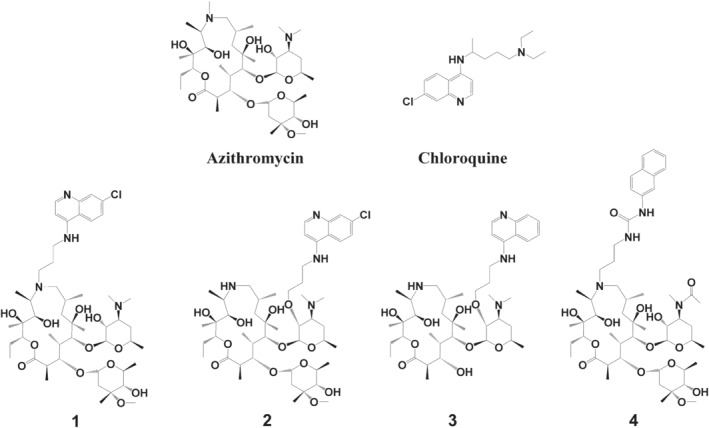
Structures of the compounds used in this study

Azithromycin (Figure 1), a 15‐membered macrolide antibiotic belonging to the semi‐synthetic azalide subclass, is characterized with a broader spectrum of antibacterial activity, improved PK/PD properties, and safety profile (approved for use in children ≥6 months of age) than its forerunners (Schönfeld & Kirst, 2002).

Macrolide antibiotics inhibit protein translation, acting through binding to the 50S subunit of the prokaryotic ribosome (Hansen et al., 2002). Since the discovery of the antimalarial activity of azithromycin (Gingras & Jensen, 1992), extensive efforts in clinical trials have been invested in exploring its usefulness for the treatment (Chandra et al., 2015; Dunne et al., 2005; Krudsood et al., 2000; Krudsood et al., 2002; Laufer et al., 2012; Noedl et al., 2006; Sagara et al., 2014; Thriemer et al., 2010) as well as prevention of malaria (Andersen et al., 1998; Kimani et al., 2016; Luntamo et al., 2010; Phiri et al., 2016; Unger et al., 2016; van den Broek et al., 2009). Azithromycin mainly inhibits the growth of asexual forms of *Plasmodium* strains in the blood. The target of its antimalarial mode of action is in the apicoplast, a plant‐like organelle, where it binds to the prokaryote‐like ribosomes and inhibits protein synthesis (Dahl & Rosenthal, 2007; Sidhu et al., 2007). This antimalarial mode of action exerts a slow killing effect on the parasites known as “a delayed death phenotype” (Fichera & Roos, 1997). Through this mode of action, azithromycin shows a marked increase in in vitro potency upon prolonged drug exposure (from 48 to 96 h). Although clinical evaluation showed the safety and usefulness of azithromycin in malaria, its future as a new antimalarial agent is uncertain as strong confirmation for the equivalence or superiority to other antimalarials is lacking (Rosenthal, 2016; van Eijk & Terlouw, 2011).

In the recent years, progressively challenging requirements are set up for new antimalarial drug candidates. Ideally, new antimalarial compounds should show fast and high reduction in parasite load, therapeutic efficacy after ideally a single dose, oral bioavailability (F), activity against all known clinical strains, block parasite transmission, prevent relapse, be safe in adults, children, and during pregnancy plus providing affordable therapy for the patients in the developing countries (Burrows, van Huijsduijnen, Mohrle, Oeuvray, & Wells, 2013; malERA Consultative Group on Drugs, 2011; Wells, Alonso, & Gutteridge, 2009). Azalides, as a class of compounds, have the optimal potential to generate the lead drug candidates with most of such desirable properties for the treatment and/or prophylaxis of malaria (Paljetak et al., 2017), particularly showing promising long half‐life suitable for single‐dose treatments. In this work, we present results from the discovery efforts of fast‐acting and potent 15‐membered antimalarial azalides (Bukvic et al., 2011; Peric et al., 2012; Pesic et al., 2012; Starcevic et al., 2012), focusing on the profiling of the four representative compounds generated within this class. The compounds presented here (Figure 1) are the main leads (compounds **1**, **2**, and **3**) resulting from our discovery program showing the most balanced potency and drug metabolism and pharmacokinetics (DMPK) properties. Compound **4** is presented and discussed because of its intriguing mode of action.

## 2 METHODS

### 2.1 Compounds

**SCHEME 1:**
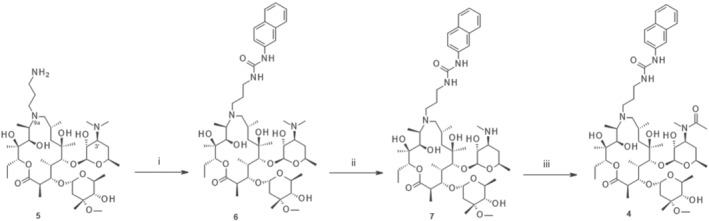
The synthesis of compound **4**. Reagents and conditions: i, 2‐naphthyl isocyanate, DCM, room temperature, 1 h, yield = 89%; ii, I_2_, MeOH, 500‐W lamp for 2.5 h then room temperature, 24 h, yield = 83%; iii, acetyl chloride, TEA, DCM, room temperature, 4 h, yield = 63%

Compounds **1**, **2**, **3**, and **4** are proprietary compounds synthesized by GSK Medicinal Chemistry in Zagreb, and the synthesis of **1**, **2**, and **3** has been described previously (Peric et al., 2012; Pesic et al., 2012). The synthesis of compound **4** is schematically presented in Scheme 1. The key intermediate, 9a‐(γ‐aminopropyl) derivative **5** was prepared by Michael addition with excess of acrylonitrile and catalytic hydrogenation with PtO_2_ according to previously published procedures (Bukvic et al., 2011; Peric et al., 2012; Pesic et al., 2012; Starcevic et al., 2012). *N*″‐2‐naphthyl‐substituted 9a‐(*N*′‐carbamoyl‐γ‐aminopropyl) derivative **6** was obtained by the reaction of corresponding isocyanate with amine **5** in high yield (yield 89%). Functionalization of the desosamine sugar was performed by demethylation of C‐3′‐dimethylamino group following the protocol described in Starcevic et al. (2012) (Bukvic et al., 2011; Peric et al., 2012; Pesic et al., 2012). The iodine‐promoted demethylation in MeOH by 500‐W lamp irradiation was used for demethylation of derivative **6** and gave a very high yield of compound **7** (yield 83%). Subjecting intermediate **7** to acetyl chloride in dichloromethane resulted in the formation of derivative **4** (yield 63%).

### 2.2 In vitro studies

#### 2.2.1 Antimalarial assay

The activity of the test compounds against *P. falciparum* in vitro was determined using the tritiated hypoxanthine incorporation assay of Desjardins et al. and additionally modified by Milhous and Chulay (Chulay, Haynes, & Diggs, 1983; Milhous, Weatherly, Bowdre, & Desjardins, 1985). Strains with the following resistance patterns were used: 3D7A (sensitive), W2, K1, V1/S, 7G8 (chloroquine /pyrimethamine resistant), FCR3 (chloroquine /atovaquone resistant), T9/94 (chloroquine resistant), Dd2 (chloroquine /quinine/pyrimethamine/sulfadoxine resistant), and in vitro generated strain Dd2(AZ^R^) harbouring a mutation in the apicoplast‐encoded *P. falciparum* ribosomal protein L4 gene (*Pfrpl4*) making it resistant to azithromycin (Sidhu et al., 2007).

Compounds were dissolved in DMSO and subsequently diluted in the culture medium (RPMI 1640) supplemented with 10% v/v human plasma. Serial 1:2 drug dilutions were prepared in triplicate in microtitre plates. Culture medium supplemented with 10% v/v human plasma and type A Rhesus positive human erythrocytes infected with *P. falciparum* strains was added to yield a haematocrit of 3% and a parasitaemia of 0.25–0.55%. The plates were incubated at 37°C in a modular incubator flushed with a mixture of 5% oxygen, 3% carbon dioxide, and 92% nitrogen for 48 h, and then [^3^H]hypoxanthine was added to give a final concentration of 12.5–16 μCi·ml^−1^. After incubation for additional 24 h, particulate matter was harvested on fibreglass strips and hypoxanthine incorporation determined by scintillation spectrophotometry. From the concentration–response curves analysed by non‐linear regression, the 50% inhibitory concentrations (IC_50_) for each test compound were calculated and the presented IC_50_ represent mean values of at least three independent experiments with SDs usually being 10–20% and not exceeding 33%, which is standard for this type of assay. Delayed death experiments were performed by this standard method but extending the incubation time with the drug up to 96 h.

#### 2.2.2 Cytotoxicity assay

The detailed protocol for cytotoxicity measurement is described elsewhere (Verbanac et al., 2005). In brief, HepG2 cells were maintained in complete RPMI 1640 medium supplemented with 10% FBS at 37°C in a 5% CO_2_ atmosphere. Each culture in the 96‐well plates contained 50,000 cells which were exposed to serial dilutions (1:2) of compounds initially dissolved in DMSO and subsequently diluted in supplemented RPMI 1640 medium. Compounds were tested in duplicates, and plates were incubated for 24 h at 37°C in 5% CO_2_. The cytotoxicity assay was performed using the MTS CellTiter 96 AQueous One Solution Cell Proliferation Assay (Promega, USA). After the addition of MTS reagent and 2 h of incubation at 37°C in 5% CO_2_, the absorbance at 490 nm was recorded and Tox_50_ determined based on the obtained response curves.

#### 2.2.3 Accumulation and retention in human cells

The human biological samples were sourced ethically, and their research use was in accord with the terms of the informed consents under an IRB/EC approved protocol. Human erythrocytes were isolated from fresh whole blood. Blood cells were removed from the plasma by centrifugation at 1,650 g and 4°C for 15 min. The pellet was re‐suspended in saline and centrifuged at 3,000 g and at room temperature for 10 min. Supernatant and the upper part of pellet containing leukocytes were discarded. The procedure was repeated three to four times until there were no traces of leukocytes. The quality of erythrocyte isolation was determined on Sysmex SF‐3000 haematological analyser. To measure compound accumulation in cells, erythrocytes (2 × 10^9^) were re‐suspended in 3‐ml RPMI 1640 (Gibco, Invitrogen) medium containing 20 μM of tested compounds and incubated at 37°C for 3 or 24 h. Cells were subsequently washed twice in ice‐cold PBS (Sigma) followed by centrifugation at 3,000 g and 4°C for 10 min. The cells were lysed by freezing in 0.5% Triton X‐100 (Sigma) in deionized water. To determine retention in cells, erythrocytes were firstly loaded with tested compounds (20 μM, 3 h), washed in ice‐cold PBS, and then incubated in pure RPMI medium for the next 0.5 or 3 h. Cells were then washed and lysed as described above. Concentrations in lysates were determined by LC–MS/MS. Samples were diluted 10‐fold in 0.5% Triton X‐100. Preparation of standards for calibration curve, sample preparation for LC–MS/MS, and analysis were carried out as described previously (Munic, Kelneric, Mikac, & Erakovic Haber, 2010), except that roxithromycin was used as an internal standard. To determine intracellular concentration, measured concentration in lysates was normalized to the volume of erythrocyte pellet in samples.

Accumulation and retention in human primary cells was determined as described in detail previously (Stepanic et al., 2011). Briefly, normal human bronchial epithelial cells (NHBE, CC‐2541, Lonza), normal human lung fibroblasts (NHLF, CC‐2512, Lonza), and bronchial smooth muscle cells (BSMC, CC‐2576, Lonza) were cultivated according to the protocols of the suppliers. Human polymorphonuclear cells (PMN) and monocytes from healthy volunteers were isolated from buffy coats. Monocytes were isolated by negative selection on magnetic separator. Monocyte‐derived macrophages (MDM) were obtained by cultivating monocytes with 5 ng·ml^−1^ rhGMCSF for 10 days. To determine compound accumulation cells were incubated with 3–10 μM of compounds in their corresponding culture medium for 3 h at 37°C in 5% CO_2_, washed, and lysed. To measure cellular retention of compounds, after being washed, drug‐loaded cells were incubated in fresh medium for 3 h, washed, and lysed. Compound concentrations were determined by HPLC–MS/MS analysis and expressed as % of azithromycin measurements.

#### 2.2.4 Antibacterial screen

Antibacterial activity of compounds was determined by a standard broth microdilution method (Clinical Laboratory Standard Institute CLSI, 2009), except that for *Streptococcus* strains lysed blood was substituted with 5% horse serum in the testing medium. Azithromycin and chloroquine were used as controls. The activities are expressed as minimum inhibitory concentrations (MICs) in units of μg·ml^−1^. The compounds have been tested in at least two and up to five independent experiments generating same or similar results (a range of one to two dilutions between tests was considered acceptable which is in accordance to CLSI acceptable quality control ranges). The organisms tested represented macrolide sensitive Gram‐positive pathogens that are mainly targeted by macrolide antibiotics. Tested streptococci were clinical isolates *Streptococcus pneumoniae* SP030 and *Streptococcus pyogenes* 3565 from internal strain collection, and other strains were obtained from ATCC bioresource centre, *Staphylococcus aureus* ATCC13709, *Haemophilus influenzae* ATCC49247, and *Moraxella catarrhalis* ATCC23246. Double dilutions of tested compounds were prepared using TECAN Genesis 150. Bacteria were grown on appropriate agar plates (Becton Dickinson, USA)—Columbia agar with 5% sheep blood for streptococci and *M. catarrhalis*, Mueller‐Hinton chocolate agar for *H. influenzae* , and Mueller‐Hinton agar for staphylococci.

#### 2.2.5 Haem polymerization

Experiments were performed according to Tripathi, Khan, Walker, and Tekwani (2004). Briefly, a solution of sodium acetate containing mono‐oleoyl glycol and the corresponding inhibitor at different concentrations is placed in 1.5‐ml reaction tubes. The reaction is triggered by adding a solution of haem (100‐μM final concentration), and after mixing, the tubes are incubated at 37°C overnight to allow the reaction to take place. Multiple washing steps with Tris/SDS and bicarbonate, to remove monomeric haem and haem aggregates, allow quantification of de novo formed β‐haematin.

#### 2.2.6 Metabolite identification in vitro

Metabolite identification in vitro was performed using cryopreserved rat and human hepatocytes (XenoTech, Kansas City, USA). Test compounds (10 μM) were incubated for 4 and 24 h at 37°C in supplemented DMEM containing 0.5 × 10^6^ cells·ml^−1^. The reaction was stopped by addition of 80:20 acetonitrile:methanol: 1% formic acid. Samples were analysed by LC–MS/MS and examined by ACD (Advanced Chemistry Development, Toronto, Canada) IntelliXtract software for potential metabolites.

### 2.3 In vivo studies

All animal care and experimental studies were conducted in accordance with the GSK Policy on the Care, Welfare and Treatment of Laboratory Animals and were reviewed by the Institutional Animal Care and Use Committees (GlaxoSmithKline RTP, Zagreb, and Tres Cantos Animal Care and Use Committee). In particular, for activities performed in Europe, all animal studies were ethically reviewed and carried out in accordance with European Directive 2010/63/EU and the GSK Policy on the Care, Welfare and Treatment of Animals. Animal studies are reported in compliance with the ARRIVE guidelines (Percie du Sert et al., 2020) and with the recommendations made by the British Journal of Pharmacology. The studies were designed to use groups of equal size with the minimal number of randomized animals.

#### 2.3.1 *P. falciparum* murine model

The in vivo efficacy evaluation of the compounds was performed using a *P. falciparum* murine model of malaria in non‐myelodepleted NOD. Cg‐Prkdc^*scid*^–β2m^*tm1Unc*^‐*scid IL2R*_*null* (NODscidβ2m^−/−^) mice engrafted with human erythrocytes as described previously (Angulo‐Barturen et al., 2008; Jimenez‐Diaz et al., 2005; Jimenez‐Diaz et al., 2009; Jimenez‐Diaz et al., 2009), as this model enables testing of *P. falciparum* strains with high infectious burden. Briefly, groups of three mice engrafted with human erythrocytes were infected with 20 × 10^6^ of *P. falciparum* 3D7^0087/N9^‐infected erythrocytes/mouse by intravenous route. The mice were randomly assigned to their corresponding experimental groups. The treatment of oral doses QD for 4 days started at day 3 after infection. Parasitaemia was measured at days 3, 5, and 7 post‐infection. The therapeutic efficacy was expressed as the dose (mg·kg^−1^) that reduced parasitaemia in peripheral blood of mice at day 7 after infection by 90% (ED_90_) with respect to vehicle‐treated mice. Chloroquine was included as quality control for each in vivo assay. Efficacy is expressed as the daily dose (ED_90_), or equivalent exposure (AUC_ED90_), administered to mice necessary to reduce parasitaemia at day 7 by 90% with respect to vehicle‐treated mice. The ED_90_ is estimated by fitting a four‐decade logistic equation for the log_10_ [parasitaemia at day 7] versus the log_10_ [dose] using GraphPad Prism 5.01 (GraphPad Software Inc., San Diego, CA).

#### 2.3.2 Pharmacokinetic studies in mice

For pharmacokinetic (PK) studies in mice, animals were dosed either by intravenous (i.v.) or oral (p.o.) route. For i.v. administration, the dosing volume was 10 ml·kg^−1^ for a total dose of 12.5 mg·kg^−1^, and for p.o. administration, the dosing volume was 20 ml·kg^−1^ for a total dose of 12.5 mg·kg^−1^. Dosing solutions were prepared in 100% saline (2.5 mg·ml^−1^) both for i.v. and p.o. administration for all compounds with the exception of compound **4** for which dosing solutions were prepared in PBS with 5% Cremophor‐EL (Fluka). Following i.v. dosing, blood samples were collected at 5 and 15 min, 1, 3, 6, 8, 24, and 48 h post‐dose. Following oral dosing, blood samples were collected at 15 and 30 min, 1, 2, 4, 6, 8, 24, and 48 h post‐dose.

For studies performed in Swiss albino CD‐1 mice (Harlan Laboratories, Spain), in order to obtain simultaneous blood and plasma profiles and *blood‐to‐plasma* (B/P) ratio, PK was performed using terminal sampling of *N* = 3 animals per time point and data evaluated using composite analysis. For studies performed in *P. falciparum* infected and non‐infected NODscidβ2m^−/−^ mice (Charles River Laboratories, France), only whole blood was obtained by serial sampling of *N* = 3 animals using tail puncture.

For analysis of whole blood samples, 25 μl of fresh blood were mixed with 25 μl of saponin solution (0.1% in water) and immediately frozen on dry ice. Plasma was obtained by centrifugation of fresh blood at 2,500 g for 10 min. Both blood and plasma samples were stored frozen at −80°C until analysis.

Diluted blood and plasma samples were processed under standard liquid–liquid extraction procedures using acetonitrile containing internal standard (clarithromycin, 100 ng·ml^−1^) and analysed by LC–MS/MS in positive ion mode with electrospray. Samples were assayed for parent compound using a Sciex API 4000 and Sciex API‐2000 Triple Quadrupole Mass Spectrometer (Sciex, Division of MDS Inc., Toronto, Canada), against a series of matrix matched calibration curve standards, using multiple reaction monitoring (MRM) at the specific transitions for each compound. Non‐compartmental analysis was performed using WinNonlin, version 4.1 (Pharsight, Mountain View, CA), and the main pharmacokinetic parameters were estimated.

#### 2.3.3 Pharmacokinetic studies in rats

For the in vivo pharmacokinetic study, compound **1** was dosed i.v. at 5 mg·kg^−1^ (5 ml·kg^−1^ dosing volume) and p.o. at a dose of 25 mg·kg^−1^ (10 ml·kg^−1^) to male Sprague–Dawley rats (Charles River, France), weighing ~350 g. Compounds **2** and **3** were dosed i.v. at 2 mg·kg^−1^ (5 ml·kg^−1^ dosing volume) and p.o. at a dose of 10 mg·kg^−1^ (10 ml·kg^−1^). Rats were fasted for 6–12 h (overnight fasting) prior oral administration. For serial blood sampling, rats (*n* = 3) dosed with an i.v. bolus of compound had blood collected at the following time points after dosing: 5, 10, 20 40, 60, 120, 240, 480, 1,440, and 1,800 min. Rats (*n* = 3) dosed orally had blood collected at the following time points after dosing: 15, 30, 60, 120, 240, 360, 480, 1,440, and 1,800 min.

Dosing solutions were prepared in 100% saline both for i.v. and p.o. After both routes of administration, blood samples were collected from the tail vein up to 30 h, haemolysed, and frozen until analysis. Samples were prepared for analysis by protein precipitation with six volumes of acetonitrile:methanol, 2:1 (v/v) containing internal standard and analysed by LC–MS/MS in positive ion mode with electrospray. Subsequently, samples were assayed as described for mouse PK.

#### 2.3.4 Pharmacokinetic studies in dogs and monkeys

Non‐naïve male cynomolgus monkeys or male beagle dogs were used for single‐dose PK studies and were fasted overnight prior to administration of test compound. Food was returned 4 h post‐dose, and water was provided freely throughout the studies. Dosing solutions were prepared in 0.9% saline supplemented to a final concentration of 1% acetic acid. An i.v. dose of 2 mg·kg^−1^ (dose volume of 1 ml·kg^−1^) and a p.o. dose of 10 mg·kg^−1^ were administered (dose volume of 5 ml·kg^−1^) to monkeys and dogs (three animals per dose group). After i.v. dosing, blood samples were collected at 5, 10, 15, and 30 min, 1, 2, 4, 6, 8, 24, 48, 72, 96, 144, and 168 h post‐dose. After oral dosing, blood samples were collected 15 and 30 min, 1, 2, 4, 6, 8, 24, 48, 72, 96, 144, and 168 h post‐dose. Blood samples were stored at −80°C until analysis and were assayed as described for mouse PK.

### 2.4 Data analysis

The data and statistical analysis comply with the recommendations of the *British Journal of Pharmacology* on experimental design and analysis in pharmacology. Data collected from animal studies include analyses on samples collected from three animals, so we consider the analysed data to have exploratory value. Declared group size is the number of independent values, and data analysis was done using these independent values. No outliers were identified in reported experiments, and no data were excluded from analyses. The methodology used for data analysis for each experimental design is described in detail in the specified sections above.

### 2.5 Materials

Azithromycin and chloroquine were supplied by USP (Rockville, USA) and [^3^H]hypoxanthine was supplied by PerkinElmer (Boston, USA).

### 2.6 Nomenclature of targets and ligands

Key protein targets and ligands in this article are hyperlinked to corresponding entries in the IUPHAR/BPS Guide to PHARMACOLOGY (http://www.guidetopharmacology.org).

## 3 RESULTS

The four compounds belonging to structurally different azalide classes were tested and their activities compared to azithromycin and chloroquine, the two main chemical “building blocks” used to construct the novel compounds (Figure 1). Compounds have a 4‐amino chloroquinoline moiety covalently linked through a propyl amine chain to the azalide scaffold: at 9a‐*N* position in compound **1** and at 2′‐*O*‐position in compounds **2** and **3**. Additionally, the cladinose sugar was removed from the 15‐membered azalide scaffold in compound **3**. Compound **4**, as one of the most active non‐chloroquinoline compounds detected in the screening process, was chosen to test beyond the azithromycin – chloroquine paradigm and provide a proof‐of‐concept for the choice of the azalide scaffold as the basic building block. The synthesis of compound **4** is presented in Scheme 1.

### 3.1 In vitro activity

Antimalarial activity of compounds was initially determined in *P. falciparum* , 3D7A (sensitive), and W2 (chloroquine/pyrimethamine resistant) strains (Table 1). After 72 h of incubation, compounds demonstrated similar antimalarial potency against both strains (ranging from 5 to 40 nM) with potencies increased more than 1,900‐fold over azithromycin and 62‐fold over chloroquine. Also, the activity/toxicity window (the ratio between *P. falciparum* IC_50_ and HepG2 Tox_50_s) showed that the inhibitory concentrations on *P. falciparum* growth were 1,000‐ to 7,500‐fold lower than concentrations inhibiting the growth of the human cell line.

**TABLE 1:** In vitro activity of compounds **1**, **2**, **3**, and **4** (in μM): IC_50_ against *Plasmodium falciparum* strains 3D7A (sensitive) and W2 (chloroquine/pyrimethamine resistant) after 72 h of incubation, *N* = 3; inhibition of human hepatocellular liver carcinoma HepG2 cell line (cytotoxicity), *N* = 2; and inhibition of chloroquine target in a cell‐free haem polymerization assay, *N* = 2

Compound	*Plasmodium falciparum* IC_50_ ^a^		Tox_50_	IC_50_
	3D7A	W2	HepG2	Haem polymerization
Azithromycin	11.513^b^	2.68^b^	203^b^	>50
Chloroquine	0.016^b^	0.431^b^	116^b^	3.8–10.6
**1**	0.020	0.027	30^c^	2.5–4.0
**2**	0.006	0.007	45^b^	2.2–4.0
**3**	0.020	0.027	83^b^	5.0–6.2
**4**	0.013	0.019	38	46.3

^a^ SDs were 10–33%.

^b^ Taken from Pesic et al. (2012).

^c^ Taken from Peric et al. (2012).

**FIGURE 2:**
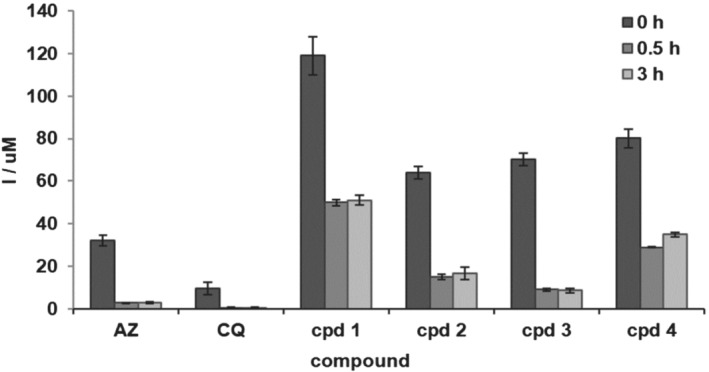
Intracellular concentration of compounds **1**–**4** in human erythrocytes compared to azithromycin (AZ) and chloroquine (CQ) with and without washout. Cells were loaded with 20 μM of compounds for 3 h, and intracellular concentration (I) was measured immediately (0 h, maximum accumulation, dark grey bars) or after a washout period of 0.5 (medium grey bars) or 3 h (light grey bars), using LC–MS/MS. Data are presented as mean values of triplicate samples with SD indicated

As macrolide antibiotics have the ability to concentrate within the host cells (Bosnar, Kelneric, Munic, Erakovic, & Parnham, 2005), accumulation and retention in human red blood cells was assessed. Within 3 h of incubation, compounds accumulated in erythrocytes in concentrations 2–3‐ and 7–12‐fold higher than azithromycin and chloroquine, respectively (Figure 2, 0 h). Longer incubation times (24 h) do not produce markedly higher intracellular concentrations (Figure S1) indicating fast and saturable accumulation kinetics into erythrocytes. Substantial amounts of the compounds were subsequently retained in the cells following the 0.5‐ and 3‐h washout period in the drug‐free medium. The concentrations measured after the washout were 3–19‐ and 9–50‐fold higher than for azithromycin and chloroquine, respectively, and up to 45% of the initially accumulated amount was retained in the erythrocytes compared to only 10% for azithromycin. Interestingly, in a set of primary human cells known to accumulate macrolide antibiotics, accumulation and retention of compound **1** was similar to that of azithromycin (Table S1), rather than threefold to fourfold higher as observed in erythrocytes.

### 3.2 Pharmacokinetics and in vivo efficacy in mouse models

Compounds were administered intravenously (IV) and orally (PO) to CD‐1 mice, and the pharmacokinetic (PK) data are summarized in Table 2. Following i.v. administration to CD‐1 mice, compounds **1**–**3** were characterized by a very low systemic CL, a moderate (compounds **1** and **2**) to large (compound **3**) Vss, a long half‐life, and low (compounds **1** and **2**) to moderate (compound **3**) oral F. Although the oral F was lower in comparison to azithromycin, the oral exposure measured in whole blood (DNAUC_PO_) was comparable and even higher at equivalent doses, likely due to a lower Vss. The B/P ratio was higher in comparison to azithromycin for compounds **1**–**3**, in line with the results obtained from in vitro uptake/retention experiments in the red blood cells. Compound **4** displayed a notably divergent PK profile, with the highest clearance and a low Vss leading to the shortest half‐life. Oral exposure was also very low and related to the lowest oral F observed. Also, compound **4** did not exhibit in vivo, the same erythrocyte uptake observed in vitro, with a low B/P ratio. Overall, PK profile exhibited was discouraging, and compound **4** was consequently removed from further progression to advanced in vitro and in vivo studies.

**TABLE 2:** PK parameters estimated in blood after intravenous and oral gavage (12.5 mg·kg^−1^) administration to CD‐1 mice (*N* = 3)

Compound	CL (ml·min^−1^·kg^−1^)	Vss (L·kg^−1^)	*t*_1/2_ (h)	DNAUC_PO_ ^a^ (ng·h·ml^−1^·mg^−1^·kg)	*F* (%)	B/P^b^
**1**	1.95^c^	4.42^c^	28.2	253	4	14.0
**2**	2.50	3.84	18.8	650	10	14.2
**3**	10.1	13.4	15.6	362	26	3.7
**4**	14.8	0.708	3.76	0.3	<1	0.4
Azithromycin	40.2	29.2	10.1	267	61	2.4

^a^ DNAUC calculated as AUC0 − *t* divided by the dose administered.

^b^ B/P values were calculated as the ratio of AUC0 − *t* measured in parallel in whole blood and plasma after oral administration.

^c^ % extrapolated AUC > 20%.

**FIGURE 3:**
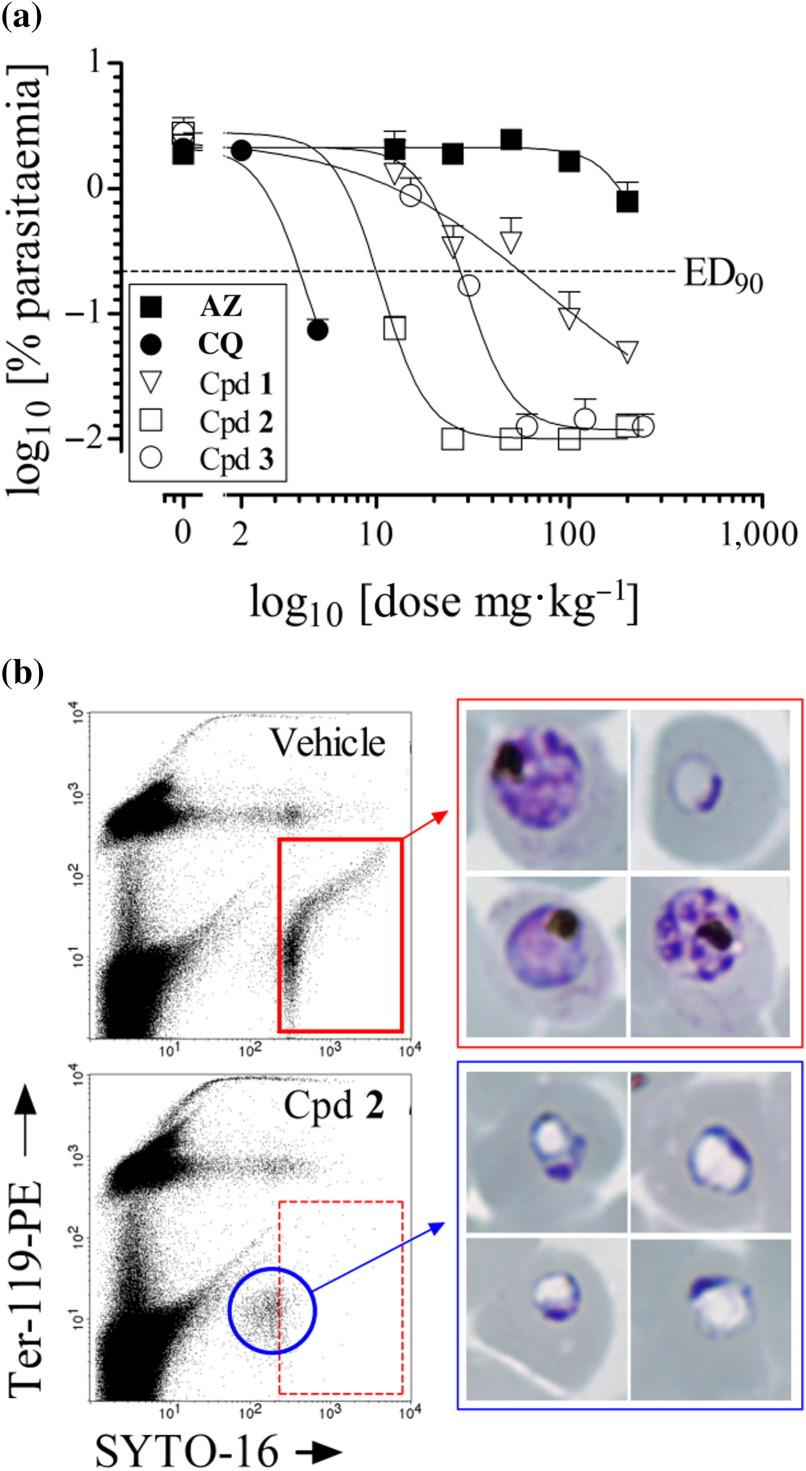
Efficacy of azithromycin (AZ), chloroquine (CQ) and compounds **1**, **2**, and **3** in *Plasmodium falciparum*‐infected NODscidβ2m^−/−^ mice engrafted with human erythrocytes. (a) Estimated ED_90_ after oral administration for four consecutive days (once daily). Data are the mean of the log_10_ [% parasitaemia at day 7] in groups of *N* = 3 mice. SD is indicated as half upper bar. (b) Analysis of *P. falciparum* in peripheral blood of mice treated with vehicle or compound **2** by microscopy and flow cytometry. Samples were taken 48 h after start of treatment (1 cycle of exposure to drug)

A humanized murine model of malaria was used to assess the efficacy of the new macrolides. The NOD‐*scid* mice engrafted with human erythrocytes allow testing the efficacy of new compounds against *P. falciparum* growing in human erythrocytes in vivo (Jimenez‐Diaz et al., 2009). Azithromycin and chloroquine were used as reference compounds: Azithromycin was not efficacious in a standardized 4‐day test (ED_90_ > 200 mg·kg^−1^) whereas ED_90_ for chloroquine was 5.1 mg·kg^−1^. The data shown in Figure 3 indicate that new azalide compounds were efficacious against *P. falciparum* 3D7^0087/N9^ (chloroquine sensitive strain) in the same mouse model, using daily oral administration. Compound **1** was the least active although it induced fast killing of *P. falciparum* parasites with only pyknotic parasites and abnormal rings observed at day 7. Furthermore, parasitaemia remained undetectable at least up to day 24 of follow‐up. Compounds **2** and **3** were more potent than compound **1** showing ED_90_ of 13.8 (11.3–16.7 mg·kg^−1^ IC 95%) and 23.9 (16.8–34.1 mg·kg^−1^ IC 95%), respectively. After treatment with compounds **2** or **3** within one parasite cycle (48 h), mostly pyknotic or aberrant haemozoin lacking trophozoites were detectable in the peripheral blood of treated mice (Figure 3b). In line with these initial microscopic findings of parasite inhibition, at day 35, two out of three mice dosed with compound **2** at 100 mg·kg^−1^ had no detectable parasitaemia in the blood sample while for the third animal, parasites were detected at day 32. chloroquine cleared parasites up to day 35 from all mice treated at 100 mg·kg^−1^. Overall, these data indicate that the new azalide derivatives had the potential to cure humanized mice infected with *P. falciparum* .

The oral exposure for compounds **1**–**3** was evaluated in *P. falciparum* ‐infected and uninfected humanized mice, along with azithromycin and chloroquine (Table 3). The DNAUCs for compounds **1**–**3** were 1.5–3.9‐fold higher in infected versus uninfected mice and 1.4–3.5‐fold higher in comparison to azithromycin. Interestingly, the intrinsic in vivo potency of compound **2** expressed as the daily molar exposure necessary to achieve 90% of parasitaemia reduction (AUC_ED90_) was 4.4 μM·h^−1^·day^−1^ which is close to the potency estimated for chloroquine of AUC_ED90_ = 3.1 μM·h^−1^·day^−1^. An additional study with compound **1** in fed versus fasted mice resulted in a threefold increase in oral F in fasted animals (Table S2), indicating that food may substantially inhibit the absorption of these compounds.

**TABLE 3:** PK parameters, mean (SD), *N* = 3, estimated in blood after oral administration to *Plasmodium falciparum* infected (I) and uninfected (U) humanized mice

Compound		*C*_max_ (ng·ml^−1^)	*t*_max_ (h)	DNAUC_(0–24)_ (ng*h·ml^−1^·mg^−1^·kg^−1^)	I/U
**CQ**	I	210 (22.5)	2.33 (0.6)	200 (13)	3.2
	U	72.3 (17.5)	1.92 (1.13)	63 (23)	
**AZ**	I	344 (133)	1.0 (0.0)	161 (7)	1.1
	U	243 (133)	1.67 (1.2)	146 (37)	
**1**	I	775 (152)	1.0 (0.0)	362 (68)	2.1
	U	173 (25)	0.75 (0.4)	169 (33)	
**2**	I	171 (3)	5.67 (4.04)	318 (25)	1.5
	U	242 (147)	4.0 (1.7)	216 (114)	
**3**	I	348 (97)	1.67 (1.16)	354 (129)	3.9
	U	83.8 (39.5)	2.2 (3.3)	91 (85)	

*Note*: Compounds were dosed at 12.5 mg·kg^−1^ and chloroquine at 10 mg·kg^−1^.

Compounds were further profiled in SD rats following i.v. and p.o. dosing, and PK parameters are summarized in Table 4. PK behaviour of compounds **1** and **3** in the rat was consistent with the profile observed in mice with low systemic CL, moderate Vss, very long half‐life, and low oral F. Compound **2** displayed a slightly higher oral F, low CL, large Vss, and longer half‐life.

**TABLE 4:** PK parameters, mean (SD), *N* = 3, estimated in blood after intravenous (2 mg·kg^−1^) and oral gavage (10 mg·kg^−1^) administration to Sprague‐Dawley rats, beagle dogs, and *Cynomolgus* monkeys

Compound	Species	CL (ml·min^−1^·kg^−1^)	Vss (L·kg^−1^)	*t*_1/2_ (h)	DNAUC_PO_ (ng*h·ml^−1^·mg^−1^·kg^−1^)	*F* (%)
**1** ^a^	Rat	2.4 (0.3)	3.8 (2.2)	23.3 (11)	169 (21)	2.3 (2.2)
**2**	Rat	4.9 (2.1)	10.8 (5.7)	30.3 (2.3)	313 (64)	7 (1)
	Dog	1.2 (0.2)	6.7 (1.0)	66.7 (2.0)	4,766 (1,259)	42
	Monkey	1.5 (0.3)	6.3 (0.7)	52.8 (11.4)	1,718 (582)	17
**3**	Rat	4.0 (0.8)	6.3 (1.8)	18.8 (1.0)	36.2 (29)	1 (1)

^a^ Compound **1** was given i.v. at 5 mg·kg^−1^ and p.o. at 25 mg·kg^−1^.

As compound **2** showed the most promising in vivo efficacy, it was selected for further profiling of its DMPK properties. Interestingly, no evidence of linker cleavage (2′‐ether linkage) or chloroquinoline functional unit modification was observed in in vitro metabolite identification experiments using rat and human hepatocytes. In addition, following i.v. administration, compound **2** had a very low blood clearance (approximately 4% liver blood flow [LBF] in the dog; 3.4% LBF in the monkey as calculated from the published LBF values for each species, Davies & Morris, 1993), large Vss, and very long half‐life of 67 and 53 h in the dog and the monkey, respectively (Table 4). The oral F was higher in comparison to rodents and was estimated to be approximately 42% in the dog and approximately 17% in the monkey.

### 3.3 Further parasitological profiling and mode of action studies

The observed high potency as well as the hybrid nature of the novel azalides motivated target‐oriented studies to better understand their anti‐plasmodial mode of action.

#### 3.3.1 Activity against a panel of chloroquine‐resistant strains

Testing of six additional chloroquine‐resistant *P. falciparum* strains (Dd2, K1, V1/S, FCR‐3, T9/94, and 7G8) was performed, and results confirmed a high level of activity across different resistance genotypes and phenotypes (Table 5). Compound **2** showed the best activity with IC_50_s being consistently low (3–6 nM) against all strains tested.

**TABLE 5:** Antimalarial activity of compounds **1**, **2**, and **3** determined against *Plasmodium falciparum* strains with different patterns and levels of resistance after 72 h of incubation (*N* = 3)

*Plasmodium falciparum* (resistance^a^)	IC_50_ (nM)^b^				
	AZ	CQ	1	2	3
K1 (CQ,PY)	2,724	313	23	5	24
FCR3 (CQ,AT)	2,590	138	22	5	22
Dd2 (CQ,Q,PY,S)	1,588	356	25	6	30
T9/94 (CQ)	2,724	178	13	3	21
V1/S (CQ,PY)	15,874	388	28	5	30
7G8 (CQ, PY)	4,586	194	37	3	17

^a^ CQ‐chloroquine, PY—pyrimethamine, AT—atovaquone, Q—quinine, S—sulfadoxine.

^b^ SDs were 10–33%.

#### 3.3.2 Antibacterial activity

As the novel azalides are based structurally on azithromycin, a successful antibiotic, the antibacterial activity of these new azithromycin derivatives was confirmed using a panel of five bacterial strains (Table S3). Compound **1** inhibited the growth of Gram‐positive bacteria (*S. aureus*, *S. pneumoniae* , and *S. pyogenes* ) in the range of azithromycin while the MICs for the Gram‐negative bacteria tested (*M. catarrhalis* and *H. influenzae* ) were higher than azithromycin. Compound **3** had no antibacterial activity while compounds **2** and **4** showed only minor activity against *M. catarrhalis*.

#### 3.3.3 Delayed death phenotype

Azithromycin manifests a reasonable in vitro efficacy as an antimalarial agent, although its true potency is only shown when parasite growth is assessed after two life cycles. This behaviour, known as the delayed death phenotype, is typical of antimalarials exerting their mode of action through inhibition of parasite organelles (Fichera & Roos, 1997). Consequently, in vitro evaluations at two different time points, 48 h (one cycle) and 96 h (two cycles), using *P. falciparum* Dd2 strain were performed. No increase in compounds' potencies was observed when the duration of drug exposure was prolonged from 48 to 96 h (Table 6). The same was shown with Dd2(AZ^R^) strain harbouring L22 ribosomal protein mutation causing azithromycin resistance (Sidhu et al., 2007) when assayed at 96 h. None of the novel azalides were affected by the described mutation, and potencies remained at a similar level for both 48‐ and 96‐h exposure time points (Table 6).

**TABLE 6:** Determination of delayed death phenotype by evaluating the potency of novel azalides against *Plasmodium falciparum* Dd2 strain and its azithromycin‐resistant mutant Dd2(AZ^R^) after 48 and 96 h of incubation (*N* = 3)

Compound	IC_50_ (nM)^a^				Delayed death phenotype
	Dd2		Dd2(AZ^R^)		
	48 h	96 h	48 h	96 h	
**A**zithromycin	3,266	81	3,359	1,485	Yes
**1**	17	12	21	11	No
**2**	10	5	10	6	No
**3**	24	22	27	23	No
**4**	65	70	68	53	No

^a^ SDs were 10–33%.

### 3.4 Cell‐free assay

Cell‐free haem polymerization assay was performed to confirm the quinoline‐like mode of action of the new compounds. Compounds **1**, **2**, and **3** inhibited haem polymerization process with even higher efficiency than chloroquine while compound **4** demonstrated ~10‐fold weaker potency (Table 1) in line with the molecular structure containing 4‐amino chloroquinoline (compounds **1**, **2**, and **3)** or naphthyl aromate (compound **4**).

## 4 DISCUSSION

Chloroquine, one of the most successful antimalarial drugs of all time in terms of its efficacy, good tolerance and low cost, has lost its previous usefulness due to the spread of resistant strains that cause recurrent treatment failures (WHO, 2015). On the other hand, azithromycin is still being tested in clinical studies as a partner drug for treatment and prevention of malaria showing attractive safety features and additional health benefits for children, pregnant women, and neonates (Gilliams et al., 2014; Porco et al., 2009; See et al., 2015; Taylor et al., 2003; Unger et al., 2015). As the right combination partner for azithromycin as well as their therapeutic niche are still under evaluation, the fate of azithromycin as a future antimalarial is not known at present (Rosenthal, 2016; van Eijk & Terlouw, 2011). In our novel drug design, we envisaged a class of compounds that would combine the best properties of these two antimalarial drugs into one hybrid molecule, not just in terms of antimalarial activity but also in overcoming resistance and taking advantage of combined physicochemical properties. Hybrid molecules are chemical entities with two or more structural domains having different biological functions, ideally dual activity, and acting as two distinct pharmacophores (Meunier, 2008). Our new hybrid molecules consist of the azalide backbone with the chloroquinoline or naphthyl aromate (a pharmacophore enhancing antimalarial activity of azalides) units bound at different positions (Bukvic et al., 2011; Peric et al., 2012; Pesic et al., 2012).

The four compounds reported here exhibited excellent in vitro activity against *P. falciparum* (low nM range) improving their potency 2–3 orders of magnitude over azithromycin (Table 1). Moreover, these compounds demonstrated consistently high potency against an array of resistant strains with various resistance patterns, including CQ^R^ and AZ^R^ strains (Tables 5 and 6).

Consequently, in the *P. falciparum* ‐infected mice, compounds **1**, **2**, and **3** demonstrated low effective doses and quick parasiticidal effect (pyknotic or aberrant trophozoites were detected already in the first parasite cycle). Interestingly, azithromycin did not show efficacy after 4‐day oral administration (two parasite cycles) in *P. falciparum* ‐infected humanized mice. These results contrast with those from previously reported animal malaria models (Andersen et al., 1995; Gingras & Jensen, 1993) but are more in line with the slow mode of action and weak efficacy shown by azithromycin in humans. The in vivo efficacy of the most active compound (compound **2**) is comparable to the efficacy of chloroquine (ED_90_ of 5.1 and 13.8 mg·kg^−1^, respectively), especially when efficacy is expressed as the molar exposure necessary to successfully reduce parasitaemia by 90% (AUC_ED90_ 4.4 and 3.1 μM·h^−1^·day^−1^ for compound **2** and chloroquine, respectively) in the model of *P. falciparum* malaria. Also, the follow‐up of parasite recrudescence in the blood of the animals showed that compound **2** suppresses parasite growth at least up to day 32, suggesting a high potential for curing malaria in humans. Furthermore, the compounds produce an in vivo rate of clearance similar to chloroquine and characteristic of fast‐acting antimalarials.

The PK analysis in mice (Tables 2 and 3) revealed that despite the lower oral F, the blood exposures of the compounds **1**–**3** were quite high, presumably a consequence of their low clearance and long half‐life. Compound **2** demonstrated consistently very long half‐lives, low clearances, and moderate oral F across species with oral exposures in the range or higher than azithromycin (Girard et al., 1987). Such PK properties might ensure infrequent dosing (once daily or even single‐dose cure) and better compliance to therapy. Also, the new compounds exhibited superior metabolic stability across species, with no products of metabolism observed for compound **2** in cryopreserved hepatocytes.

The uptake and retention in human erythrocytes revealed that compounds are abundantly accumulated and retained in these cells to a much higher extent than azithromycin and chloroquine (Figure 2). This was supported by the in vivo PK data measuring B/P ratios where the accumulation in the whole blood was up to 14‐fold higher than in plasma (Table 2) leading to the increase of the compound's concentration directly at the site of parasite infection. Also, in the infected *P. falciparum* humanized mice, oral exposures in the blood were higher than the exposures seen for azithromycin and chloroquine, demonstrating their additional capacity to accumulate in parasitized blood, for example, infected erythrocytes, and ensuring that sufficient amounts are available for the eradication of parasites after oral treatment. As it was previously shown that the critical molecular properties for cellular accumulation and retention of a basic macrolide are its lipophilicity and charge (Stepanic et al., 2011), we propose that for these new azalides the increased number of positively charged centres and the introduced aromatic rings in the scaffold increase the alkaline (cationic) as well as lipophilic properties, compared with those of azithromycin. Consequently, this enhances their ability to cross cellular membranes and become protonated at physiological pH. The gradient between the cytosolic pH of erythrocytes (7.1–7.3) and the pH of plasma (7.4) could, thus, lead these cationic amphiphilic compounds (CADs) towards the erythrocyte cytosol and, following intracellular protonation, trap them inside the cell (Funder & Wieth, 1966; Kaufmann & Krise, 2007; Stepanic et al., 2011). The same mechanism could also lie behind the additional compound accumulation observed in infected blood through further sequestration into the parasite acidic food vacuole (pH 5.4). Physicochemical properties of our novel azalides are apparently quite unique and specific since the high accumulation observed for the uninfected and infected erythrocytes does not translate into other cell types (Table S1) and remains at the level of azithromycin, thus potentially minimizing the risk of CAD‐associated side effects and maximizing parasite targeting.

Furthermore, studies of the molecular mechanisms underlying the activity of the novel azalides provided deeper insights into two assumed mechanisms of action: inhibition of prokaryotic protein synthesis (present in the parasite apicoplast and targeted by azithromycin; Sidhu et al., 2007) and haemozoin crystallization inhibited by 4‐aminoquinolines (Roepe, 2009). The assessment of the macrolide mode of action by testing for their antibacterial activity revealed that compound **1** inhibited the growth of whole cell bacteria while the other three compounds had no antibacterial activity, thus indirectly pointing to the lack of ribosome targeting in compounds **2**–**4**. It was previously shown that the antibacterial potency could be eliminated by modifying the 2′‐OH position of the azalide scaffold with the quinoline substituents, as this position is of high importance for target binding and this modification leads to the disruption of the compound‐ribosome steric complementarity (Pesic et al., 2012; Schlunzen et al., 2001).

Interestingly, the lack of delayed death phenotype in *P. falciparum* and retained potency against azithromycin‐resistant strains for all compounds implied an antimalarial mode of action distinct from that of azithromycin. This prompted us to carry out the haem polymerization test to evaluate inhibition of haemozoin formation, and the results demonstrated that the compounds with chloroquinoline substituents (compounds **1**, **2**, and **3**) inhibit haem polymerization with potencies equivalent to chloroquine. As chloroquine efficacy as well as resistance mechanisms involve complex and not fully understood biochemistry and physiology of haem chemistry and membrane transport (Roepe, 2009), so the similarities and differences of novel azalides' mode of action could only be implied here. The superior activity against an array of chloroquine‐resistant strains suggested the possibility that the hybrid nature of novel azalides contributed to parasite membrane trafficking distinct from that of chloroquine, along with a potential bypassing of the chloroquine resistance pathways mediated mainly by the mutations in the chloroquine resistance transporter PfCRT.

Overall, the results indicate that compound **1** acts potentially through at least two modes of action: (i) the more pronounced, inhibition of haem polymerization and (ii) in the background, inhibition of the apicoplast ribosome. However, because the macrolide mode of action is slow and takes effect in the second cycle, it would definitely be overridden by the fast‐acting aminoquinoline killing effect in the first parasite cycle. In compounds **2** and **3**, the macrolide‐like mode of action was eliminated, and aminoquinoline‐like mode of action remained as the sole antimalarial effect confirmed. Although the overall results from mode of action studies suggested inhibition of haem polymerization as the primary antimalarial mode of action for compounds **1**, **2**, and **3**, the properties of compound **4** have disclosed new prospects. As this compound showed a much weaker inhibition of haem polymerization and no evidence of the delayed death phenotype, while still exhibited fast in vitro antimalarial activity, comparable to other three compounds, it is conceivable that as yet unknown mode of action might also play a role in the activity of this compound, but also of other novel azalide derivatives (Bukvic et al., 2011; Peric et al., 2012; Pesic et al., 2012; Starcevic et al., 2012).

In conclusion, we propose that the novel substituted 15‐membered azalides studied here, achieve their anti‐plasmodial activity by combining and complementing their chemical, biological, and pharmacological characteristics: (i) hybrid molecule strategy contributing to the favourable physicochemical features, (ii) the mechanism of action shifting from slow to fast parasite elimination, and (iii) favourable pharmacokinetic properties (longer half‐life, lower clearance, and increased accumulation into blood compartment and even more into infected erythrocytes) combined with excellent metabolic stability (no compound degradation nor parent compound action) enabling fast and efficient eradication of *P. falciparum* parasites after oral dosing. Moreover, chemical development studies of compound **2** revealed the crystalline form as non‐hygroscopic, solvent‐free, highly soluble in biorelevant media, and physicochemically stable for up to 4 weeks in solid and dissolved state (Filic et al., 2011).

The presented results demonstrate that the described macrolide derivatives have excellent efficacy and encouraging drug‐like properties. This will facilitate their progression through the drug development pipeline, as well as the search for appropriate drug partner(s). This new therapy could provide successful new medicines in different malaria‐related indications with a high likelihood of wide use. Additional work in preclinical development and safety profiling is needed in order to fully explore the potential of these compounds and progress this class as a novel antimalarial treatment option.

## AUTHOR CONTRIBUTIONS

M.P. designed research, analysed and interpreted data, and wrote the paper; D.P. designed research and performed research; S.A. designed research and performed research; A.F. designed research and performed research; E.H. designed research, performed research, and wrote the paper; F.J.G. designed research, analysed data, and wrote the paper; I.A.‐B. performed research and wrote the paper; M.B.J.‐D. performed research and analysed data; S.F.‐B. designed research, performed research, analysed and interpreted data, and wrote the paper; M.S.M. performed research and analysed data; D.G. designed research and analysed data; A.M. performed research and analysed data; A.K. performed research; M.Ba. performed research and analysed data; J.P. designed research, analysed data, and wrote the paper; V.B.M. performed research and analysed data; V.M.K. performed research, analysed and interpreted data, and wrote the paper; M.Bu. wrote the paper; V.E.H. analysed and interpreted data and wrote the paper; R.S. designed research and interpreted data. All authors are accountable for accuracy and integrity of the presented results and have given their approval of the version to be published.

## CONFLICT OF INTEREST

F.J.G., S.F.‐B., M.S.M., and A.K. are employees of GSK, and other authors declare that they have no conflict of interest and no commercial associations that might pose a conflict of interest in connection with the submitted article.

## DECLARATION OF TRANSPARENCY AND SCIENTIFIC RIGOUR

This Declaration acknowledges that this paper adheres to the principles for transparent reporting and scientific rigour of preclinical research as stated in the *BJP* guidelines for Design & Analysis, and as recommended by funding agencies, publishers, and other organizations engaged with supporting research.

5

## Supporting information


**Figure S1.** Accumulation of compounds **1**, **2**, **3** and **4** in human erythrocytes compared to azithromycin and chloroquine. Cells were incubated with 20 μM of compounds for 3 and 24 h, and intracellular (I) and extracellular (E) concentration were measured by LC–MS/MS. Results are mean values of triplicate samples with SD indicated and presented as the I/E ratio.
Table S1. Accumulation and retention of compound **1** in human primary cells expressed relative to azithromycin, measured in the same experiment. Polymorphonuclear leukocytes (PMN), normal human bronchial epithelium (NHBE), normal human lung fibroblasts (NHLF), bronchial smooth muscle cells (BSMC) and monocyte derived macrophages (MDM). Mean values of minimum three experiments with SD in parentheses are given.
Table S2. Pharmacokinetic parameters estimated in blood for compound **1** after oral gavage (12.5 mg/kg) administration to fed and fasted CD‐1 mice (N = 3) calculated from averaged individual concentration profiles.
Table S3. Antibacterial activity of compounds **1**, **2**, **3** and **4** in comparison to azithromycin and chloroquine against bacteria sensitive to the action of macrolide antibiotics. Representative values of 2–5 independent measurements.

## Data Availability

Data are available on request from the authors.
