# Supplementary material for: A novel class of fast‐acting antimalarial agents: Substituted 15‐membered azalides
Source: Br J Pharmacol. 2020 Dec 16;178(2):363–77. doi: 10.1111/bph.15292 (PMC9328652; doi:10.1111/bph.15292)
Supplement: Supplementary file 1 — Figure S1. Accumulation of compounds 1, 2, 3 and 4 in human erythrocytes compared to azithromycin and chloroquine. Cells were incubated with 20 μM of compounds for 3 and 24 h, and intracellular (I) and extracellular (E) concentration were measured by LC–MS/MS. Results are mean values of triplicate samples with SD indicated and presented as the I/E ratio. Table S1. Accumulation and retention of compound 1 in human primary cells expressed relative to azithromycin, measured in the same experiment. Polymorphonuclear leukocytes (PMN), normal human bronchial epithelium (NHBE), normal human lung fibroblasts (NHLF), bronchial smooth muscle cells (BSMC) and monocyte derived macrophages (MDM). Mean values of minimum three experiments with SD in parentheses are given. Table S2. Pharmacokinetic parameters estimated in blood for compound 1 after oral gavage (12.5 mg/kg) administration to fed and fasted CD‐1 mice (N = 3) calculated from averaged individual concentration profiles. Table S3. Antibacterial activity of compounds 1, 2, 3 and 4 in comparison to azithromycin and chloroquine against bacteria sensitive to the action of macrolide antibiotics. Representative values of 2–5 independent measurements. [file BPH-178-363-s001.docx]

**Supporting information**

**Novel class of fast acting antimalarial agents: substituted 15-membered azalides**

Mihaela Perić^a,d,l^, Dijana Pešić^a,e^, Sulejman Alihodžić^a,e^, Andrea Fajdetić^a,e^, Esperanza Herreros^b^, Francisco Javier Gamo^b^, Iñigo Angulo-Barturen^b, f^, Mª Belén Jiménez-Díaz^b, f^, Santiago Ferrer-Bazaga^b^, María S. Martínez^b^, Domingo Gargallo-Viola^b,g^, Amanda Mathis^c, h^, Albane Kessler^b^, Mihailo Banjanac^a,e^, Jasna Padovan^a,e^, Vlatka Bencetić Mihaljević^a,e^, Vesna Munic Kos^a,j^, Mirjana Bukvić^a,e^, Vesna Eraković Haber^a,e^, Radan Spaventi^a,k^

^a^ GlaxoSmithKline Research Centre Zagreb Ltd., Prilaz baruna Filipovića 29, 10000 Zagreb, Croatia

^b^ GlaxoSmithKline, Tres Cantos Medicines Development Campus, Diseases of the Developing World, Severo Ochoa 2, 28760 Tres Cantos (Madrid), Spain

^c^ GlaxoSmithKline, Five Moore Drive, Research Triangle Park, North Carolina 27709-3398, USA

^d^ Present address: University of Zagreb School of Medicine, Center for Translational and Clinical research, Department for Intercellular Communication, Šalata 2, 10000 Zagreb, Croatia

^e^ Present address: Fidelta Ltd., Prilaz baruna Filipovića 29, 10000 Zagreb, Croatia

^f^ Present address: The Art of Discovery, Biscay Science and Technology Park; Astondo Bidea, BIC Bizkaia building, nº 612; Derio 48160, Bizkaia, Basque Country, Spain

^g^ Present address: ABAC Therapeutics, Joan XXIII, 10; E-08950 Esplugues de Llobregat; Barcelona. Spain

^h^ Present address: BioCryst Pharmaceuticals, 4505 Emperor Blvd., Durham, NC 27703

^j^ Present address: Swetox, Karolinska Institutet, Unit of Toxicology Sciences, Forskargatan 20, 151 36 Södertälje, Sweden; Karolinska Institutet, Institute of Environmental Medicine, Box 210, 171 77 Stockholm, Sweden

^k^ Present address: Triadelta Partners Ltd, Međimurska 19/2, 10090 Zagreb, Croatia

^l^ To whom correspondence should be addressed: E-mail: mihaela.peric@mef.hr.

Tel: 385-1-4590070, fax: 385-1-4566724.

**Figure S1.** Accumulation of compounds **1**, **2**, **3** and **4** in human erythrocytes compared to azithromycin and chloroquine. Cells were incubated with 20 µM of compounds for 3 and 24 h, and intracellular (I) and extracellular (E) concentration were measured by LC-MS/MS. Results are mean values of triplicate samples with SD indicated and presented as the I/E ratio.

**Table S1.** Accumulation and retention of compound **1** in human primary cells expressed relative to azithromycin, measured in the same experiment. Polymorphonuclear leukocytes (PMN), normal human bronchial epithelium (NHBE), normal human lung fibroblasts (NHLF), bronchial smooth muscle cells (BSMC) and monocyte derived macrophages (MDM). Mean values of minimum three experiments with SD in parentheses are given.

|  | **% azithromycin** | |
| --- | --- | --- |
|  | **accumulation** | **retention** |
| **PMN** | 127 (36) | 101 (19) |
| **NHBE** | 162 (3) | 123 (11) |
| **NHLF** | 100 (11) | 85 (3) |
| **BSMC** | 148 (77) | 118 (19) |
| **MDM** | 118 (21) | 94 (0) |

**Table S2**. Pharmacokinetic parameters estimated in blood for compound **1** after oral gavage (12.5 mg/kg) administration to fed and fasted CD-1 mice (N=3) calculated from averaged individual concentration profiles.

|  | **C _max_**  **(ug/ml)** | **t _max_**  **(hr)** | **AUC_0-t_ (ug*hr/mL)** | **F**  **(%)^a^** |
| --- | --- | --- | --- | --- |
| **fed** | 0.162 | 2.0 | 3.16 | 3.75 |
| **fasted** | 0.582 | 2.0 | 9.51 | 11.3 |

^a^ Bioavailability calculated as the ratio of the estimated AUC_0-t_ values after PO and IV administration.

**Table S3.** Antibacterial activity of compounds **1**, **2**, **3** and **4** in comparison to azithromycin and chloroquine against bacteria sensitive to the action of macrolide antibiotics. Representative values of 2-5 independent measurements.

|  | **MIC (μg/ml)** | | | | |
| --- | --- | --- | --- | --- | --- |
| **Cpd** | ***S. pneumoniae***  **SP030** | ***S. pyogenes***  **3565** | ***S. aureus***  **ATCC13709** | ***H. influenzae***  **ATCC49247** | ***M. catarrhalis***  **ATCC23246** |
| AZM | ≤0.125 | ≤0.125 | 1 | 2 | ≤0.125 |
| CQ | 64 | >64 | >64 | >64 | >64 |
| **1** | ≤0.125 | ≤0.125 | 1 | 16 | 2 |
| **2** | >64 | >64 | >64 | >64 | 16 |
| **3** | >64 | >64 | >64 | >64 | >64 |
| **4** | >64 | >64 | >64 | >64 | 32 |
